# Artificial intelligence in personalized rehabilitation: current applications and a SWOT analysis

**DOI:** 10.3389/fdgth.2025.1606088

**Published:** 2025-07-24

**Authors:** Elpidio Attoh-Mensah, Arnaud Boujut, Mikaël Desmons, Anaick Perrochon

**Affiliations:** ^1^ University of Limoges, HAVAE UR 20217, Limoges, France; ^2^ 3iL Ingénieurs, XR Laboratory, Limoges, France

**Keywords:** artificial intelligence, personalization, rehabilitation, SWOT, healthcare, patient-centered approach

## Abstract

Artificial intelligence (AI) is transforming personalized rehabilitation by introducing innovative methods to enhance care across diverse medical specialties. Despite its potential, widespread implementation remains limited, largely due to a lack of comprehensive analyses on its benefits and barriers. This mini narrative review examines current applications of AI in personalized rehabilitation and provide a SWOT (Strengths, Weaknesses, Opportunities, Threats) analysis AI is already being used to develop personalized treatment plans, support ongoing patient management, and adapt therapy sessions in real-time. One of its key strengths is the capacity to process vast datasets and monitor real-time information, thereby elevating the level of personalization. Automation of certain tasks can reduce human error and alleviate clinician workload, allowing more time for direct patient care. Opportunities for AI lie in leveraging rapidly advancing technologies to meet the rising demand for rehabilitation services, particularly with aging populations. Collaborations with industry can accelerate innovation, while data sharing can promote best practices across institutions. However, notable challenges persist. High implementation costs, ethical concerns such as algorithmic bias, and risks of increasing healthcare disparities remain major barriers. Additionally, threats such as data privacy breaches and security vulnerabilities emphasize the need for robust, balanced regulatory frameworks. In conclusion, AI holds immense promise for transforming personalized rehabilitation. While current applications are largely in early stages or proof-of-concept phases, ongoing research, ethical foresight, and strategic collaboration are essential to maximize benefits and minimize risks for optimal patient outcomes.

## 1 Introduction

Artificial intelligence (AI) is transforming the field of personalized rehabilitation by offering innovative approaches to enhance care delivery across a broad spectrum of medical specialties (1–4). AI is often defined as a machine's ability to perform tasks considered intelligent by human standards, replicating behaviors such as perception, reasoning, learning, planning, and prediction (5). By advancing the performance of AI models, substantial opportunities arise in the field of rehabilitation, including mitigating health disparities (6), enhancing diagnostic precision, and optimizing communication with healthcare providers (7, 8), thereby fostering more equitable and efficient healthcare ecosystems (9). Numerous literature reviews highlighted the use of AI in various rehabilitation programs demonstrating significant potential to enhance effectiveness (10–15).

AI techniques applied in rehabilitation can be broadly categorized into machine learning and deep learning. Machine learning detects patterns in data to make predictions, with some models improving over time, while deep learning specializes in recognizing complex patterns like images and speech using layered neural networks (16). Examples of AI applications in rehabilitation include analyzing datasets to identify patterns, such as the link between exercise intensity and recovery speed (17, 18). One can also make predictions or integrate real-time therapy data to adapt to patients' progress, optimizing rehabilitation outcomes dynamically (19).

Notably, personalizing rehabilitation based on individual patient characteristics can be an interesting application of AI in healthcare (17, 20, 21). Personalization in rehabilitation is particularly relevant since research consistently demonstrates that the more a program is tailored to a patient's unique profile and needs, the more effective it becomes (22, 23). For example, personalization can be needed when one should align with the patient's capabilities in exercise prescription, thereby enhancing adherence and minimizing injury risk (24). The personalized approach further improves patient outcomes and lowers healthcare costs, fostering more sustainable healthcare systems (25–27). That being said, it is essential to distinguish between individualization and personalization. Individualization refers to tailoring programs to a specific need for patients considering objective assessments such as medical diagnoses, functional limitations, and goals. In contrast, personalization goes further with a patient-centered approach, integrating their views and objectives into the program (28). While both individualization and personalization are applied in rehabilitation, personalization involves a higher level of complexity due to the need to interpret and integrate many data sources. This is where the utility of AI can be invaluable, with several advantages (e.g., processing a large amount of data, task automation, error reduction…, etc.).

A SWOT analysis critically evaluates the strengths, weaknesses, opportunities, and threats associated with a given phenomenon. This analytical tool, well-regarded across multidisciplinary fields (29–31), offers a comprehensive overview while highlighting key strategic dimensions. In the context of utilizing AI in rehabilitation, it could serve as a valuable resource for guiding future research and shaping clinical applications. Despite a few examples in health fields such as medical education, surgery (30, 32), or sports (31), a SWOT analysis in personalized rehabilitation is still lacking.

The present mini review followed a narrative approach with a two-fold objective. First, it aims to provide an overview of the current applications of AI in personalized rehabilitation, offering new insights. Second, it presents a SWOT analysis to evaluate the strengths, weaknesses, opportunities, and threats associated with the use of AI in developing personalized rehabilitation programs.

## 2 Current applications of AI in personalized rehabilitation

**Figure 1:**
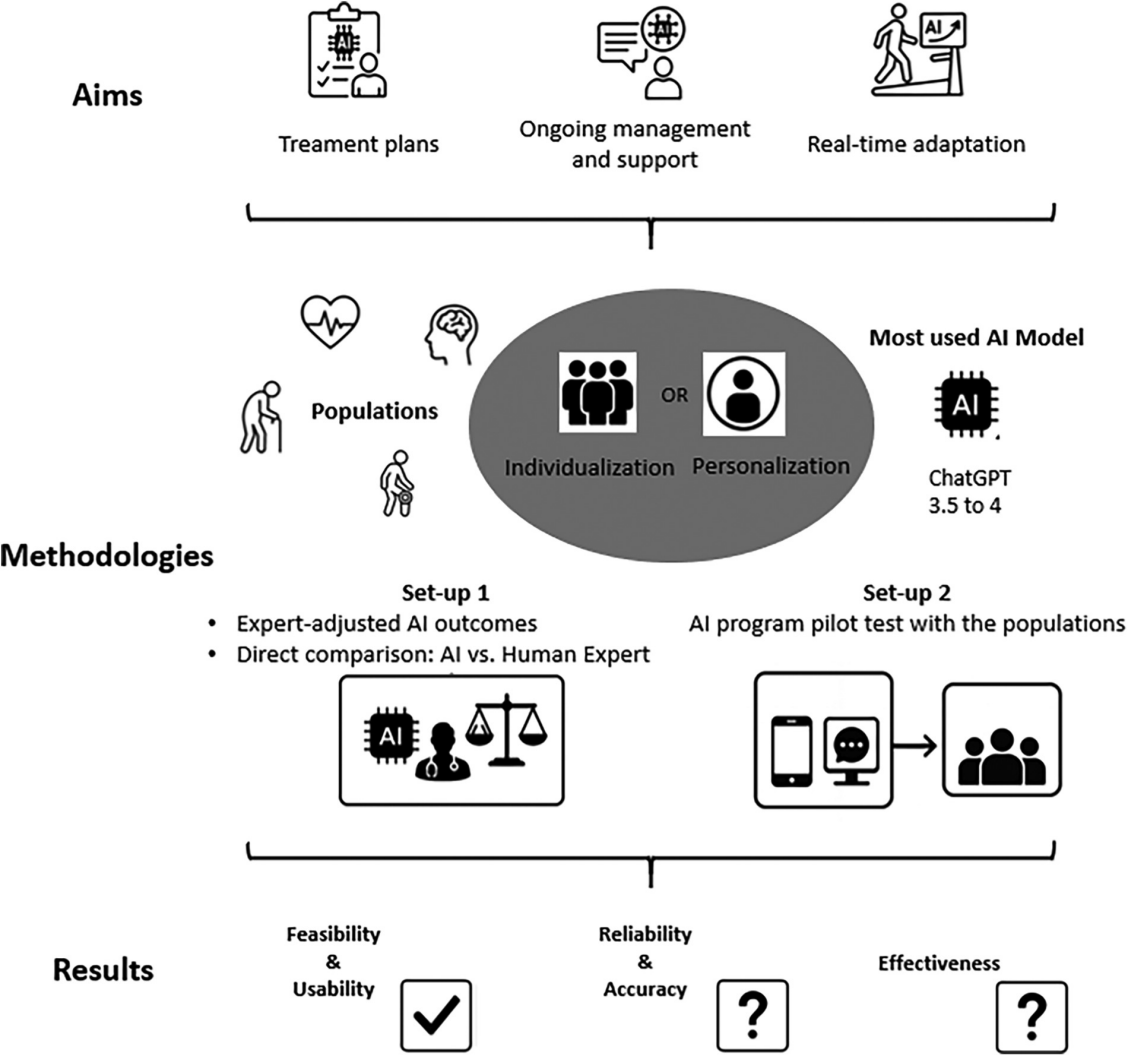
Current applications of artificial intelligence in personalized rehabilitation. Flowchart depicting AI applications in healthcare, focusing on treatment planning, ongoing management, and real-time adaptation. Aims include personalization and individualization using ChatGPT models. Methodologies cover expert-adjusted outcomes, AI-human expert comparisons, and pilot testing. Results reflect feasibility, usability, reliability, accuracy, and effectiveness; categories marked with question marks indicate insufficient data.

As illustrated in Figure 1, AI in personalized rehabilitation is used across diverse patient populations, including those recovering from strokes, musculoskeletal disorders, chronic pain, or even cardiac and neurocognitive conditions (17, 33, 34). Notably, ChatGPT (versions 3.5 to 4) emerged as the primary AI tool employed across these applications. Noteworthy, the studies often addressed individualization rather than personalization in the sense that the characteristics mainly were relevant to the patients’ pathology (17, 33–37) rather than the personal insights and preferences of the patient (19, 38). In addition, the studies were protocol papers, case studies, observational, cross-sectional or remained at an exploratory stage, except for a few trials that have investigated the effectiveness of AI, particularly ChatGPT, in enhancing the personalization of rehabilitation (39, 40). In our context, several studies have contributed data across each of the three following areas: to generate treatment plans (17, 33, 41, 42), provide ongoing management and support, such as delivering up-to-date information on diseases (35–37, 43), and to dynamically adapt therapy sessions by functioning as a just-in-time adaptive digital assistant (19, 38, 44). Noteworthy, while the ChatGPT system has been reported to provide useful, safe, and valid information for patient management, with agreement rates among physicians ranging from 55% to 80% (35, 36), it is important to note certain limitations. In particular, the accuracy of generating specific treatment plans or real-time adaptation remains a concern, with performance reaching only about 70% (19, 42).

AI can be used to design personalized treatment plans by analyzing large datasets, including patient histories and profiles. Some studies investigated the effectiveness of ChatGPT in generating rehabilitation plans tailored to diverse populations and contexts (34, 39, 40, 45). Additionally, several studies assessed ChatGPT's performance by comparing its rehabilitation recommendations with those of expert physiotherapists, evaluating its potential to provide low-risk treatment plans (17, 41, 42). Such studies have found that the AI model can create general safety-conscious exercise programs for various scenarios, although it still lacks precision in addressing individual health conditions and goals (17, 41).

Second, AI can not only assist in creating these plans but also in managing them throughout the rehabilitation process, offering ongoing support to enhance personalization over time. In this category, ChatGPT functioned as a chatbot, offering guidance and support to patients undergoing rehabilitation (35–37, 43). This usage investigated in the studies yielded satisfaction in motivating users to keep doing their physical activity program (43), or to provide patients with relevant information on their disease and available rehabilitation plans (35–37). Importantly, there may be some limitations regarding the comprehensiveness, accuracy, and readability of the generated recommendations in this chatbot usage (35, 37).

Finally, AI can be used as a just-in-time adaptive assistant by analyzing live data collected during sessions, allowing for dynamic adjustments tailored to the patient's immediate needs and performance. ChatGPT was explored in one study for its potential to generate adaptive content to help parents support their child's physical activity level (44). This study showed that ChatGPT effectively identified feedback previously validated within a framework (46), addressing the intention-behavior gap, which encompasses ongoing reflexive and regulatory processes essential for a given activity (44). Other studies used inertial measurement units and other trackers to capture body movements and activity during rehabilitation (19, 38). Machine learning algorithms were then applied to these data to accurately adjust the training dosage, enabling more precise and personalized therapy (19, 38).

Overall, it is clear that AI holds significant promise for personalized rehabilitation. However, its integration into clinical practice can still be challenging because obstacles and barriers need to be clearly identified, as discussed below in the SWOT analysis (4).

## 3 SWOT analysis

### 3.1 Strength

Personalization of rehabilitation programs requires the processing of diverse patient-centered data, which can be challenging. AI is a powerful tool to address this challenge by handling large amounts of data to create highly tailored rehabilitation programs (31). Recent studies have demonstrated the feasibility of using AI to develop rehabilitation interventions based on socio-demographic data and physical functioning (17, 34, 45). This advantage of AI could be taken further by integrating more personal data (e.g., patient preference, inclusion of lifestyle, adherence to activity of daily life…) to increase the level of personalization. This ability to include high level of personalization has the potential to enhance the overall effectiveness of rehabilitation. Tailored programs improve the precision of interventions, increase patient engagement, and optimize recovery outcomes by addressing individual barriers and goals (22, 23).

Furthermore, AI could enhance personalization by dynamically adapting real-time rehabilitation therapy based on patient profiles. Indeed, by integrating real-time data from wearable sensors, motion tracking systems, and physiological monitors, AI can analyze a patient's responses during therapy sessions and make immediate modifications to optimize the rehabilitation process (19). The real-time adaptability will allow for precise adjustments based on movement quality, fatigue levels, and neuromuscular responses. This ensures that each session is tailored to the patient's physical state, which can fluctuate daily. Such an approach enables a more responsive and efficient rehabilitation experience, potentially accelerating recovery and improving long-term outcomes (1, 31).

Healthcare personnel are often overwhelmed by their workloads, which can result in patients receiving incorrect or suboptimal rehabilitation programs (47–49). This mismanagement can lead to poor patient adherence, rendering ineffective interventions (50, 51). AI can help mitigate the workforce issue by automating specific time-consuming tasks (e.g., patient initial assessment and monitoring, administrative tasks) (1). As a consequence, it can reduce human error and improve the precision of rehabilitation programs. Studies comparing AI-generated predictions of high-quality rehabilitation programs with the ground truth provided by healthcare professionals have yielded encouraging results, suggesting that AI can be just as effective as humans (52). AI's capacity to automate the technical and data-driven components of the rehabilitation process could further represent a positive leverage for human resources optimization. Indeed, healthcare professionals would have more time to engage with patients on a deeper and more personal level (53). Considering the patient in healthcare remains essential, particularly in personalized rehabilitation, where motivation, emotional support, and personalized attention can significantly influence patient outcomes (54). As a result, clinicians devoting more attention to the emotional and psychological aspects of care could foster stronger relationships with patients and tailor interventions to their specific needs (55, 56). Additionally, since most rehabilitation teams are multidisciplinary, coordinating all professionals within an optimal framework for care delivery can often be challenging. An AI can help overcome this challenge, facilitating seamless collaboration and supporting safe, high-quality teamwork (57). For example, automating the scheduling of interventions within a unified platform could enable all professionals to share and track the patient's progress, ensuring coordinated care. Such improvements are likely to enhance overall service quality, leading to better outcomes and higher levels of patient satisfaction.

### 3.2 Weaknesses

One of the main obstacles to implementing AI is the cultural shift required for the adoption, which necessitates changes in behavior from both patients and healthcare professionals. This shift will likely encounter resistance from some users, potentially slowing the adoption process (58). This resistance may stem from various factors, including a lack of familiarity with AI technologies, fear of being replaced, concerns about the accuracy and reliability of AI systems, or discomfort with altering long-established workflows (2). To address these barriers, it is crucial to implement targeted efforts focused on education and training for healthcare providers. These initiatives aim to demonstrate the tangible benefits of AI in improving patient outcomes, streamlining workflows, and enhancing clinical decision-making. Providing hands-on training sessions and access to user-friendly AI tools can help build practitioners' confidence and competence. Additionally, fostering an open dialogue that addresses ethical concerns, data privacy, and the importance of human oversight in AI systems can help alleviate skepticism. Resistance to adoption is critical to consider as it is systematically presented as a weakness of AI in the SWOT analyses across various fields (30–32). It is, therefore, essential to support healthcare professionals in understanding the functioning and limitations of AI tools and training them for their effective use.

Additionally, integrating AI may require significant costs, especially during the initial stages of implementation. However, one should view these costs as an investment in long-term efficiency (26). For instance, inadequate rehabilitation services can lead to increased costs over time (25, 27), while AI-powered programs have the potential to reduce inefficiencies and generate savings in the future. Ensuring the successful adoption of AI requires research into its economic sustainability, helping healthcare systems make informed investment decisions that balance initial costs with long-term benefits.

Integrating AI into personalized rehabilitation introduces additional complexities due to structural challenges. Most healthcare systems operate within rigid and structured frameworks, which may lack the flexibility to accommodate new technologies (6). Successful implementation necessitates expertise in understanding the technical of AI tools. Specialized training is thus required to provide this expertise to healthcare professionals, but this remains challenging and demands careful planning. Indeed, balancing the allocation of time, personnel, and resources for training while ensuring continued patient care will add supplementary strain to the overall implementation process. Furthermore, even in a system where AI technology has been fully implemented and is operational, challenges related to the tool's interoperability may still emerge. Given the broad range of tasks that AI can handle, various techniques and tools (e.g., data servers, data storage, software, sensors, smartphone-based applications…, etc.) will likely be employed. As a result, integrating these tools into a unified framework can present a significant challenge.

Finally, potential errors in AI performance may still occur. These errors could stem from various factors, such as limitations in the training data, algorithmic biases, or unexpected technical malfunctions depending on the accuracy of the AI. In addition, in the context of personalized rehabilitation, the data are diverse. When these multiple input data are of poor quality or not fully compatible with the AI system, they may lead to errors or inaccurate outcomes. Such inaccuracies might lead to incorrect assessments, inappropriate recommendations, or even adverse outcomes in rehabilitation processes. Ensuring the reliability and accuracy of AI implementation is crucial to minimize risks and maintain patient safety while maximizing the potential benefits of AI-driven interventions. Human oversight is crucial to review and refine AI-delivered rehabilitation programs, ensuring that recommendations are continually fine-tuned and adapted as needed (17).

### 3.3 Opportunities

In the coming years, significant advancements in AI are expected, particularly in speed, processing power, and overall capabilities (16). As these technologies evolve, there is a unique opportunity to advance the development of AI tools and implement innovative solutions that foster their optimal use. This is especially important because, as previously noted, studies continue to rely heavily on ChatGPT in current usage of AI in personalized rehabilitation. By integrating cutting-edge AI advancements into rehabilitation practices, we can create more dynamic, effective, and tailored approaches to patient care (14). This period of rapid growth in AI presents an ideal time for healthcare professionals to harness its full potential, driving meaningful improvements in rehabilitation outcomes and overall healthcare delivery. In addition, AI is becoming increasingly accessible, with continuously improving algorithms and models optimized for deployment on portable devices such as tablets and smartphones. Emerging solutions like TinyLlamab (59) and versions of Mistral are making AI integration more feasible. Additionally, the rise of open-source AI models (60) facilitates local implementation within hospital settings, allowing institutions to leverage AI capabilities without needing external cloud-based services.

Demographic trends suggest that the demand for rehabilitation services will increase in the coming years, driven by an aging population and the rising prevalence of chronic conditions (61). The diversity of such conditions will enhance the need to personalize rehabilitation. However, the healthcare workforce required to meet these demands is not expanding at the same rate (47). This discrepancy presents a unique opportunity for AI to play a pivotal role in enhancing the delivery of rehabilitation services by automating specific tasks (31). In the context of AI-driven personalization of rehabilitation programs this would offer a continuous cycle of improvement. Indeed, as more data is collected from diverse patient populations, AI systems can refine and optimize their ability to provide tailored care.

The number of areas with limited access to care is growing (62), and this issue becomes even more critical because, when care is available, it often lacks a personalized approach due to the shortage of healthcare providers (63). AI has the potential to bridge the healthcare access gap, particularly in underserved or rural areas, by facilitating rehabilitation services that can be accessed remotely (1). For example, in exercise prescription, AI can generate personalized rehabilitation programs at distance and monitor patient progress in real-time by analyzing data from various sensors (1). This approach would extend the reach of healthcare professionals, ensuring that patients who might otherwise be unable to receive care can still benefit from rehabilitation, even with a personalization approach. A seamless integration with the growing field of telerehabilitation can deliver care directly to patients' homes, offering personalized support based on their unique environments.

AI in personalized rehabilitation opens new opportunities for collaboration with various fields. First, industries can design, develop, and refine innovative solutions that enhance effectiveness. Indeed, the utilization of advanced technologies such as extended reality, serious games, and robotics is increasingly transforming rehabilitation, significantly improving patient outcomes partly because of the ability to include personalization (10–12). Interestingly, AI hold the strength to support these technologies by analyzing patient data, monitoring progress, and optimizing their application in real-time. For example, AI can adapt extended reality simulations to match a patient's evolving needs, customize serious games to maintain engagement and therapeutic effectiveness and control robotic devices for precise, personalized movements (10–13). Moreover, there is another valuable opportunity for collaboration within the research field of rehabilitation, with projects aiming to explore the underlying mechanisms of the contents of rehabilitation programs. Indeed, using AI to create personalized rehabilitation would require a critical phase of carefully selecting the most relevant data to customize the program. Since rehabilitation programs target various mechanisms that drive recovery, it is essential to know which data to consider in the personalization (e.g., individual goal, lifestyle) to effectively improve the outcomes. For instance, neuroplasticity plays a key role in post-stroke rehabilitation and must be optimally stimulated. Therefore, when using AI to deliver personalized rehabilitation, the data considered during the initial assessment should prioritize patient factors that promote better neuroplasticity. This includes elements like exercise integrated into daily activities, patient preferences, and a supportive environment tailored to the patient's lifestyle (64). Thus, this is an opportunity to develop future research aiming to understand how patient characteristics interact with rehabilitation programs to provide AI with valuable data to create the most effective personalized strategies.

Finally, AI systems could facilitate data sharing between health institutions across different countries, enhancing the accuracy of algorithms. By integrating diverse patient profiles, AI's ability to personalize care would improve significantly. Such cross-border data sharing promotes the exchange of best practices, contributing to a more unified and efficient approach to healthcare, particularly in telerehabilitation, where efforts are already underway to standardize care across borders (65). Another opportunity might also arise for a new type of role, where a dedicated professional manages data and ensures its proper integration into AI systems across institutions. This approach could address the challenge of training healthcare professionals in AI and ease the strain on resource management.

### 3.4 Threats

While AI presents significant potential, there are also essential threats, primarily related to patient data privacy and security, which have been systematically outlined in previous SWOT (30–32). Ensuring that data is collected and used solely for the patient's benefit is crucial, and strict regulations are necessary to protect patient privacy (2, 31). In addition, such data presence could further expose healthcare institutions to cyberattacks, posing an additional security challenge. However, overly stringent regulations could limit innovation and hinder the full potential of AI in healthcare. Finding the right balance between privacy and innovation would be difficult.

Another significant threat is AI's potential to amplify existing healthcare inequalities. High initial costs associated with implementing AI-driven rehabilitation programs can be a substantial barrier for low-budget medical centers, limiting their ability to adopt these technologies and widening disparities in care quality. Furthermore, the inequalities extend beyond financial constraints, raising ethical concerns about inclusivity. Algorithmic biases in AI systems, often stemming from the underrepresentation of certain demographic groups in training datasets, may lead to the exclusion of specific patient populations, exacerbating disparities in access and outcomes (2, 6).

Finally, there might be a significant expectation surrounding the role of AI in personalized rehabilitation. This could lead to personnel becoming overly reliant on technology, resulting in reduced control and limited human interaction in the process (2). Important questions arise about the clinician's role in patient care. There is a risk of over-relying on AI and mistakenly reducing the need for clinicians. Therefore, while AI can assist in prescribing rehabilitation programs, medical experts must regularly review and oversee these plans. Indeed, one should ensure that AI serves as a tool to facilitate personalized care, rather than replacing the caregiver's role, to maintain the integrity and effectiveness of the rehabilitation process.

## 4 Limitations

**Figure 2:**
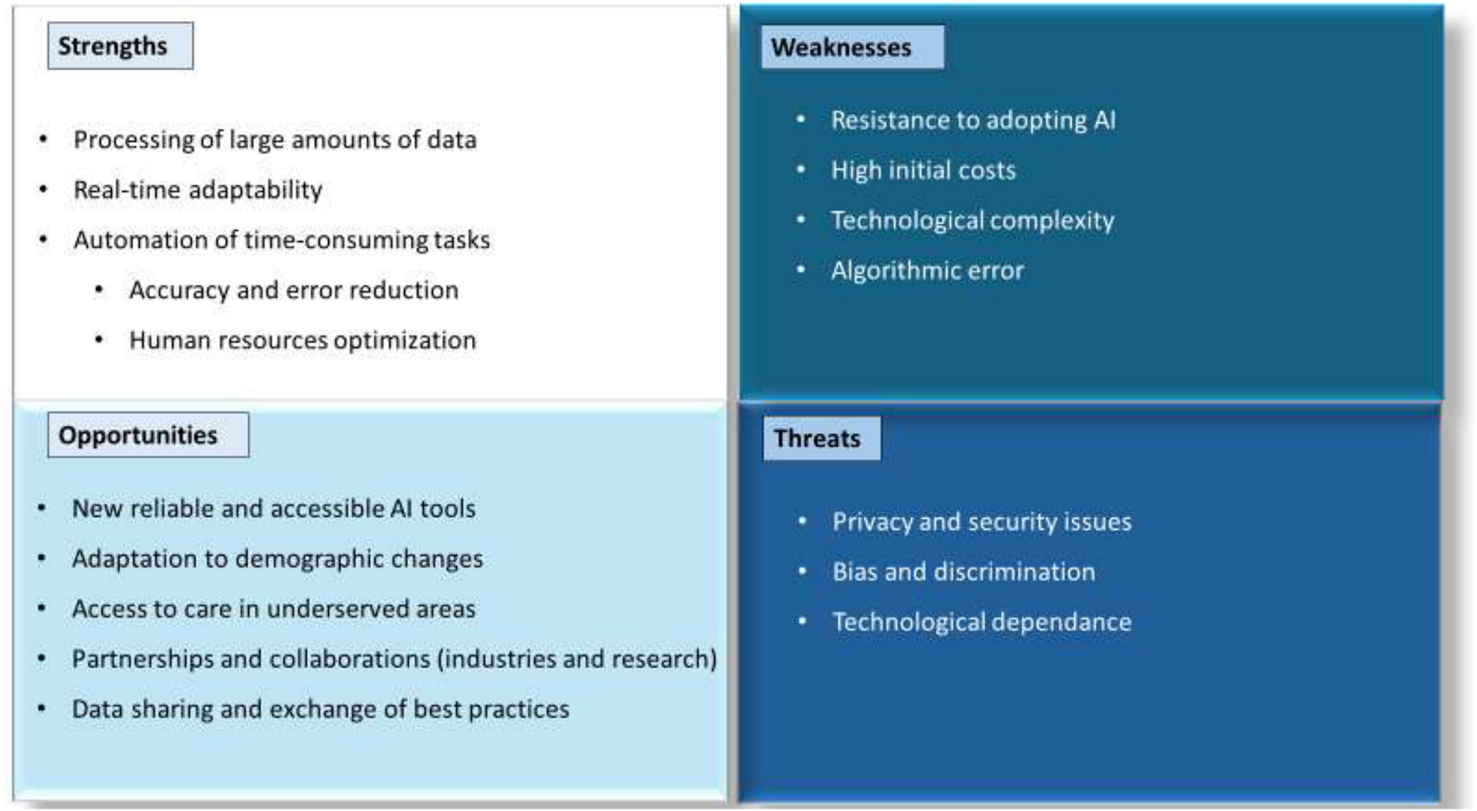
Strengths, weaknesses, opportunities and threats of the implementation of artificial intelligence in personalized rehabilitation.

One of the primary limitations of this study, which employs a SWOT analysis, is the absence of a systematic approach, raising concerns about the comprehensiveness and potential biases of the results. However, it is important to highlight that conducting a fully systematic analysis in this context remains challenging due to the evolving nature of AI applications in rehabilitation. Given the rapid advancements in AI-driven rehabilitation, achieving an exhaustive analysis at this stage would be inherently difficult. While this general approach may limit direct applicability to specific cases, it serves as a crucial starting point for future research, providing a foundational perspective from which more targeted studies can emerge. Finally, while some of the items identified in our SWOT analysis may overlap with findings from previous studies, our approach goes a step further. We highlight new items other than these recurring elements and provide a detailed examination of their relevance to personalized rehabilitation. In particular, we emphasize how each item could be specifically implemented, providing deeper insights into the use of AI in personalized rehabilitation. A summary of the key points of our SWOT can be found in Figure 2.

## 5 Conclusion

AI presents exciting opportunities to revolutionize personalized rehabilitation by enhancing care delivery and improving efficiency. Current applications range from the generation of treatment plans to the real-time adaptation of therapy sessions, through ongoing management and support. Despite the wide array of available AI technologies, many studies still rely heavily on ChatGPT as the primary tool. AI can process vast amounts of patient-specific data, enabling the creation of highly personalized rehabilitation programs that optimize recovery outcomes. By automating routine tasks, AI further reduces human error and the workload on healthcare providers, allowing them to dedicate more time to engaging with patients on a more personal level. As AI continues to evolve, new, reliable, and accessible tools will be available, creating new opportunities to design cutting-edge solutions by collaborating with industries. There is also an opportunity to meet the increasing rehabilitation demand stemming from demographic change particularly in underserved areas, by widening the reach of personalized rehabilitation. Despite all these advantages, challenges remain, including algorithmic biases, healthcare disparities, ethical issues, and regulatory concerns. Data privacy and security are critical, as AI handles sensitive patient data. While regulations are necessary, they must strike a balance to avoid limiting AI's potential. In conclusion, this SWOT analysis highlights AI's transformative potential in personalized rehabilitation while providing its main limitations. Continued research and careful consideration of these challenges are essential to ensure the safe and impactful implementation of AI technologies in healthcare.
